# Microbiota-derived 3-phenylpropionic acid promotes myotube hypertrophy by Foxo3/NAD^+^ signaling pathway

**DOI:** 10.1186/s13578-024-01244-2

**Published:** 2024-05-15

**Authors:** Penglin Li, Xiaohua Feng, Zewei Ma, Yexian Yuan, Hongfeng Jiang, Guli Xu, Yunlong Zhu, Xue Yang, Yujun Wang, Canjun Zhu, Songbo Wang, Ping Gao, Qingyan Jiang, Gang Shu

**Affiliations:** 1 State Key Laboratory of Swine and Poultry Breeding Industry, Tianhe District, 483 Wushan Road, Guangzhou, 510642 Guangdong China; 2 Guangdong Laboratory for Lingnan Modern Agricultural and Guangdong Province, Tianhe District, 483 Wushan Road, Guangzhou, 510642 Guangdong China; 3 National Engineering Research Center for Breeding Swine Industry, College of Animal Science, South China Agricultural University, Tianhe District, 483 Wushan Road, Guangzhou, 510642 Guangdong China; 4 Key Laboratory of Animal Nutritional Regulation, College of Animal Science, South China Agricultural University, Tianhe District, 483 Wushan Road, Guangzhou, 510642 Guangdong China

**Keywords:** 3-Phenylpropionic acid, Gut microbiota metabolites, Muscle hypertrophy, NAD^+^, Acetylation

## Abstract

**Background:**

Gut microbiota and their metabolites play a regulatory role in skeletal muscle growth and development, which be known as gut-muscle axis. 3-phenylpropionic acid (3-PPA), a metabolite produced by colonic microorganisms from phenylalanine in the gut, presents in large quantities in the blood circulation. But few study revealed its function in skeletal muscle development.

**Results:**

Here, we demonstrated the beneficial effects of 3-PPA on muscle mass increase and myotubes hypertrophy both in vivo and vitro. Further, we discovered the 3-PPA effectively inhibited protein degradation and promoted protein acetylation in C2C12 and chick embryo primary skeletal muscle myotubes. Mechanistically, we supported that 3-PPA reduced NAD^+^ synthesis and subsequently suppressed tricarboxylic acid cycle and the mRNA expression of SIRT1/3, thus promoting the acetylation of total protein and Foxo3. Moreover, 3-PPA may inhibit Foxo3 activity by directly binding.

**Conclusions:**

This study firstly revealed the effect of 3-PPA on skeletal muscle growth and development, and newly discovered the interaction between 3-PPA and Foxo3/NAD^+^ which mechanically promote myotubes hypertrophy. These results expand new understanding for the regulation of gut microbiota metabolites on skeletal muscle growth and development.

**Supplementary Information:**

The online version contains supplementary material available at 10.1186/s13578-024-01244-2.

## Introduction

As a crucial component of human body, skeletal muscle not only plays a major role in locomotor activity but also in metabolic homeostasis, energy consumption and protein storage [1]. Skeletal muscle development can be simply divided into three parts: the proliferation and the formation of myofibers, myotube hypertrophy and the repair of myotube injury, which are subject to the regulation of energy homeostasis, muscle angiogenesis [2], protein turnover and so on. The hypertrophy process jointly regulated by multiple signaling pathways, among which phosphatidylinositol 3-kinase (PI3K)/AKT/mTOR is the classical signaling pathway regulating protein synthesis in skeletal muscle. And Foxo family, a classical signaling pathway in protein degradation [3], is a class of transcriptionally active regulators whose activity is influenced by numerous upstream signaling molecules and is also regulated by post-translational modifications of proteins. Moreover, Foxo3 targets serval myogenic transcription factors [4, 5] and protein degradation-related genes, and recently some new effectors with Foxo3 had appeared in promoting muscle hypertrophy [5, 6] and inhibiting muscle atrophy [7–9]. SIRT1/3 is a member of NAD-dependent deacetylases family, that could modify Foxo3 cytosolic location and regulate its binding to target genes [10, 11] to modulate cell energy expenditure [5]. Interestingly, Foxo3 can modulate SIRT/NAD^+^ at transcriptional level that formed as a feedback regulation. However, there still are few studies explored the combination function of SIRT and Foxo3 in skeletal muscle development.

Recent years the gut-muscle axis, which refers to the composition of gut microbiota and their metabolites on skeletal muscle metabolism and functionality [12], has been gained wide attention. Germ-free mice that lack gut microbiota exhibited signs of muscle atrophy, mitochondria dysfunction and metabolic mode switching in muscle [13–15]. And microbiota metabolites, such as secondary bile acids, short-chain fatty acids [16] and microbial aromatic amino acids [17–19] could improve C2C12 proliferation, skeletal muscle mass and protein metabolism by influencing the glucose uptake and mitochondria function of skeletal muscle. Among lots of microbiota metabolites, 3-phenylpropionic acid (3-PPA) metabolizes from phenylalanine which is one of essential aromatic amino acids. Through LS-MA analysis, 3-PPA be found as the third substantially produced metabolites by colon microbiota in pigs that be ranked behind short-chain fatty acid and lithocholic acid [17, 20]. At present, the reports about 3-PPA is still shallow, mainly be relevant to its function in regulating insulin secretion after the intake of glucose and jointly maintaining blood glucose balance [21, 22] via G-protein-coupled receptor 40 which can express in C2C12 myotubes. Based on those, the function of 3-PPA in regulating skeletal muscle development still unclear.

In this study, we reported that 3-PPA significantly increase the myotubes diameter by restraining protein degradation instead of protein synthesis. By using siRNA interference and immunoprecipitation, we found that 3-PPA inhibited NAD^+^ synthesis and tricarboxylic acid cycle, decreased the mRNA expression of SIRT1/3, which facilitates the acetylation of total protein and Foxo3. Moreover, we demonstrated that 3-PPA also can inhibit Foxo3 protein expression in C2C12 nuclei by direct binding through AutoDock software and CETSA assay. Overall, our findings provided a new understanding of the function and the mechanism of microbiota metabolites on skeletal muscle development.

## Materials and methods

### Cell culture

The mouse myoblast cell line C2C12 (ATCC, RRID: CVCL_0188) was cultured in high glucose DMEM (GIBCO, Grand Island, NY, USA) with penicillin (100 U/mL), fetal bovine serum (10%) and streptomycin (100 μg/mL) at 37 °C, in a humidified atmosphere containing 5% CO_2_. When cells got 90% confluency, culture media was switched by DMEM with 2% horse serum to induce myoblast differentiation to myotubes for 6 days.

### Primary chicken embryos skeletal muscle cells extraction

The leg muscle tissues of 10-embryo-old chicken embryos were collected, and the bones, mucous membranes and skin tissues were removed with surgical scissors and forceps. The muscle tissues were cut to the shape of minced meat, then digested with the addition of trypsin in the incubator at 37℃ for 15~20 min (repeat blowing for 5-6 times) and terminated the digestion with complete medium (20% fetal bovine serum and 0.5% penicillin, streptomycin in high-sugar DMEM) to terminate digestion. After filtration with a 70 μm filter, the cells were centrifuged at 1500 r/min for 5 min, resuspended in complete medium, transferred to culture dishes, and wall-applied twice to obtain purified myoblasts. The purified cells were placed in the incubator at 37 °C with 5% CO2.

### Puromycin incorporation

Myotubes were incubated with puromycin at a concentration of 1 μg/mL after the end of 3-phenylpropionic acid treatment for 1 h. At the end of the incubation, the cell plates were washed three times with PBS, and the proteins were extracted and subjected to the Western Blot assay. Exposure was performed using FluorChem M Fluorescent Imaging System (Protein Simple, San Jose, CA, USA); the images obtained after exposure were counted in grey scale by ImageJ software.

### NAD+/NADH/Acetyl-CoA/Glucose/Lactic acid content detection

The NAD+/NADH content assay kit was purchased from Beyotime Biotechnology (S0175); The Acetyl-CoA content assay kit was purchased from Solarbio(BC0980); Glucose and lactic acid content test kits were purchased from Nanjing Jianjian Bioengineering Institute (F006-1-1, A019-2-1). These assays were performed along with those kit instructions.

### Foxo3 siRNA transfection

The transfection steps and siRNA sequences of Foxo3 was described in our previous study. The siRNA of Foxo3 was purchased from Guangzhou RiboBio Co., Ltd (Guangzhou, China) and transfected with lipofectamine (Invitrogen, Carlsbad, CA, USA) followed by the manufacturer’s instructions.

### Immunoprecipitation

Immunoprecipitation was performed with Protein G Dynabeads(10003D,Thermo Fisher Scientific). The Dynabeads were reacted with rabbit anti-Foxo3 (A0102, ABclonal), Pan-acetylation (66289-1-lg, Proteintech) and Histone H3 (4499S, CST) and rabbit anti-IgG (Normal IgG, CST) for 4 h at room temperature washed with buffer solution for three times. The Dynabeads were incubated with equal amounts of total or fractionated protein extracts overnight at 4 ℃ in a vertical mixing rotator, washed with buffer solution three times, and collected the Dynabeads. The Supernatant collection is used as input control. The collected Dynabeads were denatured at 100 ℃ for 10 min with 2xSDS-PAGE buffer, and stored in – 20 ℃ until the Western blot assay as the previous methods.

### Western blot analysis

Cells or muscles were cracked by the RIPA lysis buffer containing 1 mM PMSF. For the nuclear or cytoplasmic protein extraction, the procedure of protein extraction was followed by the nuclear extraction kit (BB3112, Bestbio). Protein concentration was detected by a BCA protein assays kit. After sodium dodecyl sulfate (SDS) polyacrylamide gel electrophoresis gels, total protein lysates (20 μg) were immunoblotted with primary antibody (P-Foxo3: AP0684, ABclonal; Foxo3: A0102, ABclonal; P-mTOR: 5536S, CST; mTOR: ab185696, Abcam; P-AKT, 4071S, CST; AKT: 9272S, CST; Ubiquitin: A2129, ABclonal; Puromycin: MABE343, EMD Millipore Corporation; P-JAK2: BS-2485R, Bioss; JAK2: 3230S, CST; P-STAT3: 9145S, CST; STAT3: 12640S, CST; MyoD: sc-377460, Santa Cruz Biotechnology; MyoG: 382257, CST; MyHC: MAB4470, CST; Histone H3: 4499S, CST; Pan-acetylation: 66289-1-lg, Proteintech; GPR43: sc-293202, Santa Cruz Biotechnology; followed by incubating with goat anti-rabbit or goat anti-mouse HRP-conjugated secondary antibody (1:50 000). The levels of GAPDH, VDAC and β-actin served as the loading control. Protein expression levels were determined using MetaMorph software ImageJ (National Institutes of Health, USA).

### RNA extraction, reverse transcript, and qPCR

Total RNA from cells was extracted by using an RNA extraction kit (Guangzhou Magen Biotechnology Co., Ltd, China) and Trizol reagent (Invitrogen, Carlsbad, CA, USA) according to the manufacturer’s instructions. The total RNA was retrotranscribed into cDNA by 4xReverse Transcription Master Mix (A0010GQ) according to protocol of the kit. Using the designed primers, 2xSYBR Green qPCR Master Mix (ROX2 Plus) (A0001-R2) was used in accordance with the stated procedures. cDNA synthesis was performed with the Applied Biosystems QuantStudio 3 Real-Time PCR System. The primers sequences used for PCR are provided in Table 1.

**Table 1:** QPCR primer sequence

Gene	Forward primer sequence (5′-3′)	Reverse primer sequence (5′-3′)
DRP1	GCAACTGGAGAGGAATGCTG	CACAATCTCGCTGTTCTCGG
ATP5A1	GTTTCAACGATGGGACCGAC	TCCGTCAGTCTCTTCACCAG
COX6A1	TGCTCAACGTGTTCCTCAAG	TAAGGGTCCAAAACCAGTGC
TFAM	AGATATGGGTGTGGCCCTTG	AAAGCCTGGCAGCTTCTTTG
NDUFA6	CAAGATGGCGGGGAGCGG	GTATAGTGAGTTTATTTGTGCTC
PGC1-α	TCAGAACCATGCAGCAAACC	TTGGTGTGAGGAGGGTCATC
SIRT1	TCCTTGGAGACTGCGATGTT	AATTCCTTTTGTGGGCGTGG
PINK1	CGAGCATCTTCTAGCCCTGA	TTCTCTCTCAGCCTGTCAGC
BNIP3	TCCAGCCTCCGTCTCTATTTA	TGGTATCTTGTGGTGTCTGGG
PARK2	ACAAATGCATCTGGAGGGGA	ACCTCTGGCTGCTTCTGAAT
SQSTM1	ACATGGAGGGAAGAGAAGCC	CACCGACTCCAAGGCTATCT
ATG5	GCCATCAACCGGAAACTCAT	GATCTCCAAGTGTGTGCAGC
ATG7	GGGGTTTTCTGTCACGGTTC	AGCAGCTTGGGTCTCTTGAT
VDAC	GGCTCACCTTCACCCAAAAG	GCCGAGACTAAAACAATCCCG
Ndufb8	AAGACTACGAGCCATACCCC	GGTCCCAGTGTATCGGTTCA
SDHB	CTGTCGAGGGGCACAGAC	CAACACCATAGGTCCGCACT
NAMPT	TGATCCCAACAAAAGGTCGAA	CCCACTCACACAAAAGCCTA
NMNAT2	AGAACACCCAGCCCATTTAC	GAGGCTTTCTCCCACCTTTC
SIRT3	CAGCTACATGCACGGTCTGT	ACACAATGTCGGGTTTCACA
UQCRC1	TTCAGCAATTTAGGAACCACCC	GGTCACACTTAATTTGCCACCAA
CS	CGTTTCCGAGGTCATAGTATCCC	GCTGAGACATAGGGTGTAGGTTGG
IDH2	CCCGTATTATCTGGCAGTTCATC	ATCAGTCTGACGGTTTGG
OGDH	GCCCACCACCACTTTCATC	CCGCTTCTCCTCGTTGGT

### Immunofluorescence staining

For staining of C2C12 cell, C2C12 cell was punched in 0.4% Triton for 10 min and then blocked for 1 h with a slowly shaking at room temperature. The sections were then incubated with primary antibody at room temperature overnight in a wet box. Goat anti-rabbit FITC (bs-0295G, Bioss), goat anti-mouse IgM/Alexa Fluor 555 antibody (bs-0368G-AF555, Bioss), goat anti-rabbit Flour 555 (bs-0295G, Bioss), goat anti-mouse FITC (bs-50950, Biowarld), rabbit anti-goat IgG FITC (bs-0294R, Bioss), and corresponding second antibodies were supplied for use. A Nikon Eclipse Ti-s microscope was used to take photos of these sections. Images of fluorescent intensity were analyzed with Nis-Elements BR software (Nikon Instruments, Tokyo, Japan).

### CCK-8 and EdU incorporation Assay

An appropriate number of cells were cultured in a 96-well plate. After culturing the cell overnight and returning to normal, the required drug or other stimulation treatment was performed. Then CCK-8 detection was carried out. While for the EdU incorporation assay, cells were incubated with EdU reagent at 37 ◦C for 2 h and were then fixed in 4% paraformaldehyde for 15 min and washed with PBS 3 times (5 min each). After that, cells were permeabilized with 0.3% Triton X-100 for 10 min and washed with PBS before adding a click additive solution for 30 min. The nuclei were stained with Hoechst, and images were captured with a Nikon Eclipse Ti-s microscope (Nikon Instruments, Tokyo, Japan).

### Molecular docking

The crystal structure of the Foxo3 and GPR40 protein was downloaded from the Protein Data Bank (www.rcsb.org). The GPR40 was used as a control which is regarded as ligand of 3-PPA. The structure of 3-PPA was downloaded from ZINC (zinc.docking.org). All the steps during docking followed by the video (www.bilibili.com). We use PyMOL software to visualize the docking result and calculate the binding energy, ligand efficiency and so on.

### Cellular thermal shift assay (CETSA)

CETSA experiments were performed according to a recording protocol [23]. C2C12 cells were treated with PBS or 3-PPA. After incubation for 2 h, the cells were washed with PBS, harvested by RIPA. Protein concentration was detected by a BCA protein assays kit. Equal amounts of cell suspensions were aliquoted into 0.2 mL PCR microtubes. Subsequently, the cell suspension aliquots were heated individually at different temperatures for 3 min (GE9612T, BIO-GENER), followed by cooling for 5 min at room temperature. Then, the soluble fractions were isolated by centrifugation and analyzed by SDS-polyacrylamide gel electrophoresis (SDS-PAGE) followed by western blot as described above. Fold-changes in the immunoblot band densities (normalized to the GAPDH and using the lowest temperature condition as reference) were plotted as a function of temperature to generate foxo3 melt curves for the different treatments.

### Statistics

Statistical analyses were using GraphPad Prism 9.0 software (Chicago, IL, USA). Methods of statistical analyses were chosen based on the design of each experiment and are indicated in the figure legends. The data are presented as mean ± SEM. *P* < 0.05 was statistically significant.

## Results

### 3-PPA increases muscle mass in mice and induces hypertrophy in C2C12 myotubes

**Fig. 1:**
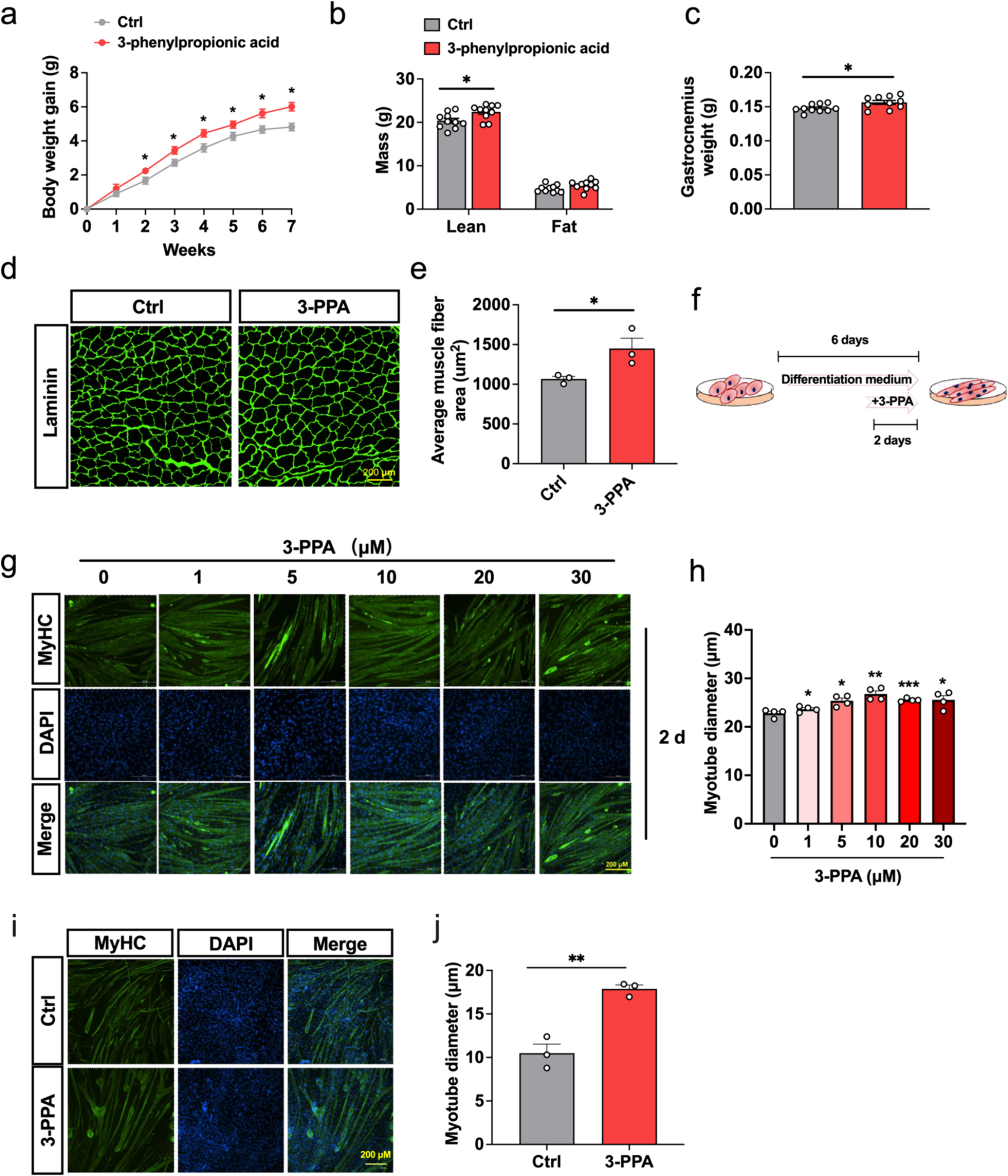
Effect of 3-PPA on muscle mass and C2C12 myotubes hypertrophy. **a** Body weight gain in 6 week-old male mice with7 weeks’ treatment of 0.5%3-PPA (n = 10). **b** Lean mass and fat mass of mice after 6 weeks’ treatment of 3-PPA by QMR (n = 10). **c** Gastrocnemius weight of mice after 7  weeks’ treatment (n = 10). **d–e** The laminin immunofluorescent staining (**d**) and frequency histogram of gastrocnemius muscle fiber cross-sectional area statistical analysis (**e**) in C57BL/6 male mice after 3 weeks 3-PPA treatment (n = 3). **f** Schematic representation of C2C12 treated with 3-PPA for 2 days. **g–h** Immunofluorescence images (**g**) and statistics of myotube diameter (**h**) of C2C12 myotubes in 3-PPA treatment for 2 days (n = 4). **i–j** Immunofluorescence images (**i**) and statistics of myotube diameter (**j**) of chick embryo primary skeletal muscle cells in 3-PPA treatment for 2 days (n = 3)

To explore the influence of 3-PPA on growth and body composition in mice, 6 week-old C57BL/6 male mice were treated with water containing 0.5% 3-PPA for 7 weeks. During this process, we found 3-PPA significantly increase the body weight gain (Fig. 1a), the lean mass and gastrocnemius weight were promoted after 6 weeks’ treatment (Fig. 1b, c). Consistently, 3-PPA can prompt muscle fiber cross-section area in Gastrocnemius muscle of mice (Fig. 1d, e). Further, we investigated the affection of 3-PPA in skeletal muscle in vitro by detecting C2C12 proliferation, differentiation and myotube hypertrophy. As shown in CCK-8 and EdU assay (Fig S1a-c), 3-PPA has no effects on the proliferation activity of C2C12 in various concentration and time. Then. we added 3-PPA in differentiation medium at differentiation day 1 and day 4 respectively. The results showed that 3-PPA significantly increase myotube diameter in 2 days treatment (Fig. 1f-h) instead of 6 days (Fig S1d-f). What’s more, 3-PPA performed the same function in chick-embryo primary skeletal muscle cells (Fig. 1i, j). Taken this together, Fig. 1 indicates that 3-PPA has important role in promoting muscle mass and myotube hypertrophy.

### 3-PPA attenuated protein degradation instead of protein synthesis in C2C12 myotubes

**Fig. 2.**
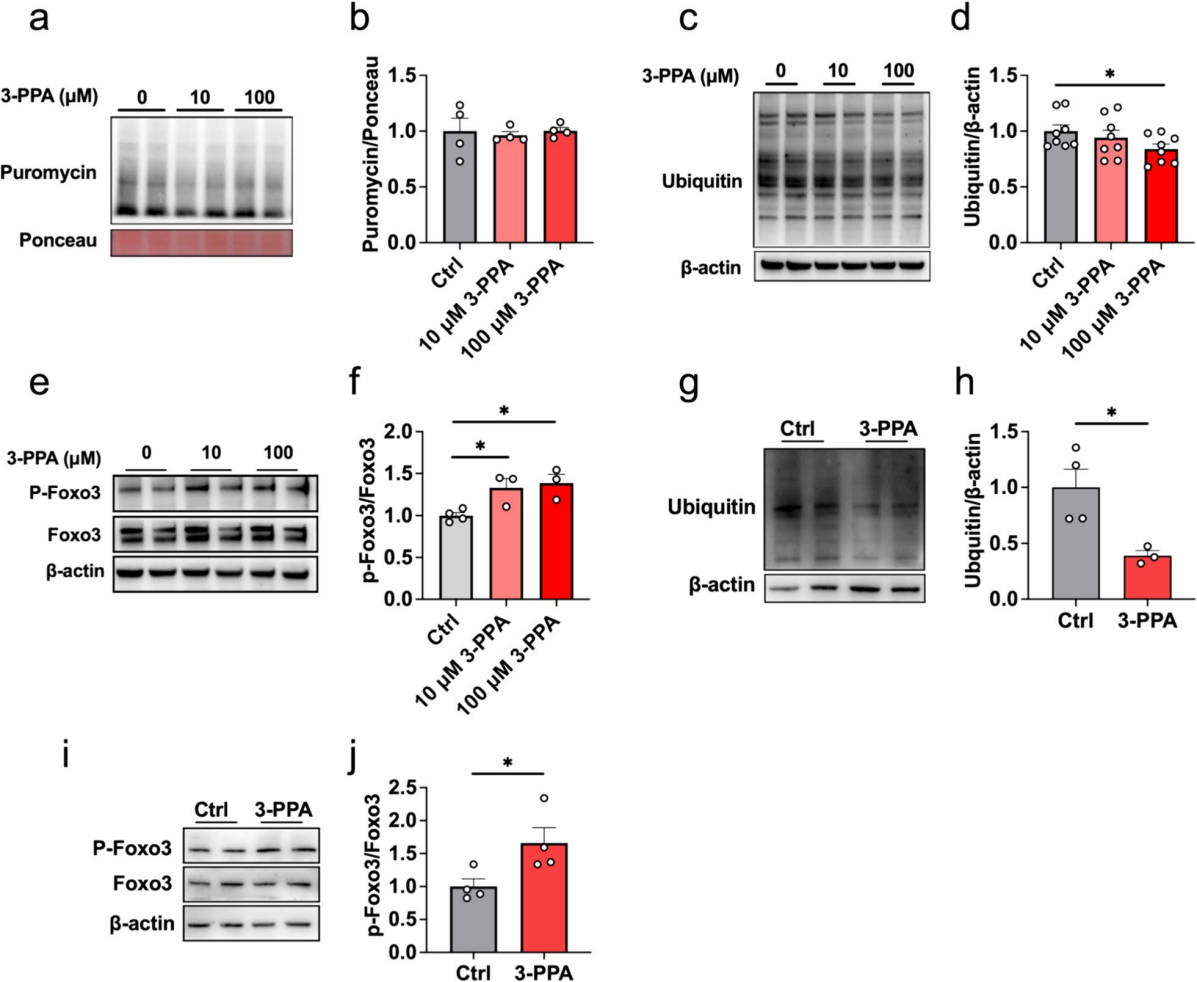
3-PPA attenuated protein degradation instead of protein synthesis in C2C12 myotubes. **a–b** Immunoblots (**a**) and quantification (**b**) of the puromycin-labeled protein expression of C2C12 myotubes with 3-PPA treatment for 2 days (n = 8). **c–d** Immunoblots (**c**) and quantification (**d**) of total ubiquitination protein expression of C2C12 myotubes with 3-PPA treatment for 2 days (n = 4). **e–f** Immunoblots (**e**) and quantification (**f**) of P-Foxo3 and Foxo3 protein expression of C2C12 myotubes with 3-PPA treatment (n = 3). **g–h** Immunoblots (**g**) and quantification (**h**) of the total ubiquitination protein expression of chick embryo primary skeletal muscle myotubes with 3-PPA treatment for 2 days (n = 3-4). (**i–j**) Immunoblots (**i**) and quantification (**j**) of P-Foxo3 and Foxo3 protein expression in chick embryo primary skeletal muscle myotubes (n = 4)

Muscle hypertrophy that performed as increased muscle nuclei and thickened muscle fibers impinge on excessive protein synthesis and reduced protein degradation [24]. To examine the mechanism of myotube hypertrophy caused by 3-PPA treatment, we firstly evaluated the 3-PPA’s effects in protein degradation and synthesis. As shown in Fig. 2c, d, we found 3-PPA suppressed ubiquitin levels of total protein, which mainly take charge of protein degradation [25], but has no effects in the expression of puromycin-labeled protein which on behalf of the rate of protein synthesis (Fig. 2a, b). Subsequently, the phosphorylation of Foxo3, which could inhibit multiple constituents of ubiquitination of protein and muscle wasting [26], was significantly increased (Fig. 2e, f). The results in chick embryo primary skeletal muscle cells were further verified the effect of 3-PPA (Fig. 2g-j). However, the protein expression of JAK/STAT3, AKT/mTOR [27] and ERK pathway which are related to regulating protein synthesis and degradation were didn’t change (Fig S2). Taken together, we found that 3-PPA has effects on inhibiting protein degradation but no protein synthesis, which may induce protein deposition and myotube hypertrophy.

### Protein acetylation modification mediates the effect of 3-PPA on myotube hypertrophy.

**Fig. 3:**
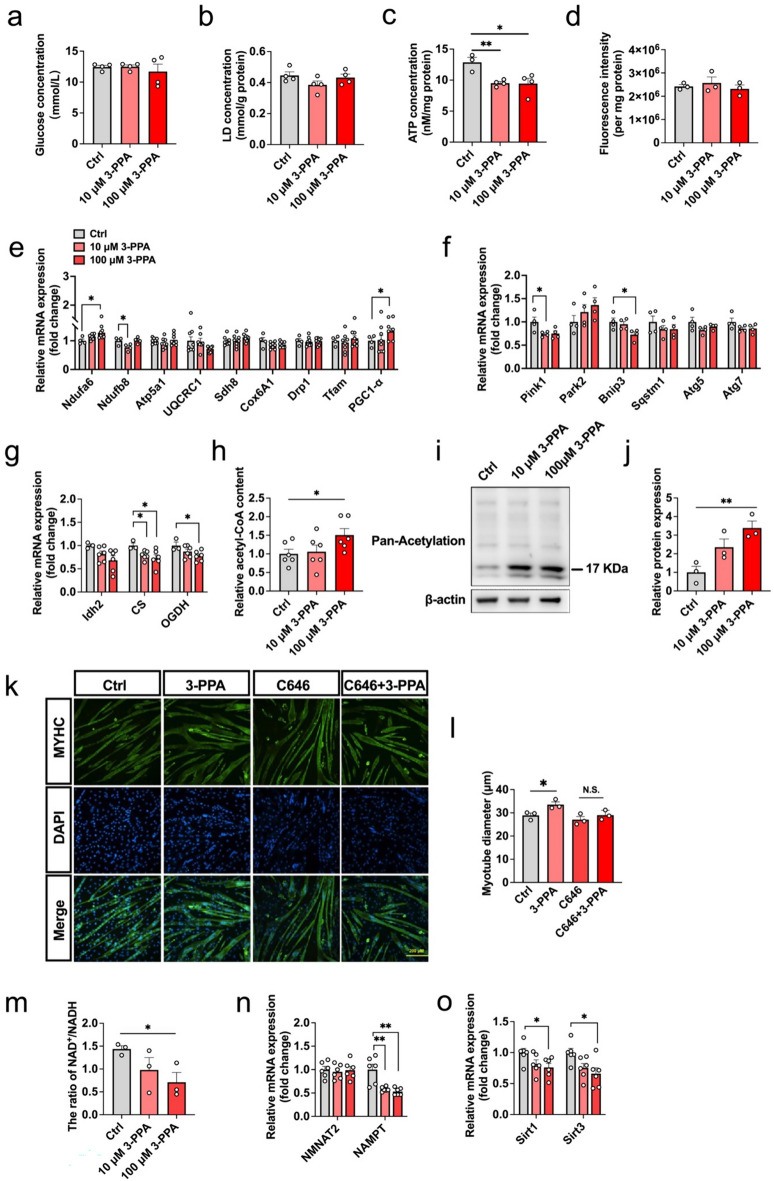
Protein acetylation modification mediates the effect of 3-PPA in myotube hypertrophy. **a–d** The glucose content (**a**), LD content (**b**), ATP content (**c**), ROS content (**d**) in C2C12 myotubes with 3-PPA treatment for 2 days (n = 3 to 4). (**e**) Mitochondria biogenesis and function related gene mRNA expression in C2C12 myotubes with 3-PPA treatment for 2 days (n = 4 to 8). **f** Mitochondria autophagy related gene mRNA expression in C2C12 myotubes with 3-PPA treatment for 2 days (n = 4). **g** Tricarboxylic acid cycle related gene mRNA expression in C2C12 myotubes with 3-PPA treatment for 2 days (n = 6). **h** Relative Acetyl-CoA content in C2C12 myotubes with 3-PPA treatment for 2 days (n = 6). **i**–**j** Immunoblots (**i**) and quantification (**j**) of Pan-acetylation protein expression of C2C12 myotubes with 3-PPA treatment for 2 days (n = 3). **k–l** Immunofluorescence images (**k)** and statistics of myotube diameter (**l)** of C2C12 myotubes in C646 and 3-PPA co-treatment (n = 3). **m** The ratio of NAD^+^/NADH in in C2C12 myotubes with 3-PPA treatment for 2 days (n = 3). **n** mRNA expression of NANAT2 and NAMPT in C2C12 myotubes with 3-PPA treatment for 2 days (n = 6). **o** mRNA expression of SIRT1and SIRT3 in C2C12 myotubes with 3-PPA treatment for 2 days (n = 6)

Since energy supply are crucial for myotube hypertrophy, we speculated that 3-PPA may promote myotube hypertrophy by affecting myotube energy metabolism. Our results showed that 3-PPA had no effect on the contents of glucose, lactic acid and reactive oxygen species which were metabolites produced by glycolysis and aerobic oxidation, but significantly reduced the content of ATP in myotubes (Fig. 3a-d). Mitochondria are the “factories” of energy creation in the body, their quantity and function directly affect the metabolic level of the body. As shown in Fig. 3e, f, 3-PPA had no effect on the expression of genes related to mitochondrial fusion and formation, but significantly inhibited the gene expression of mitochondrial autophagy markers (PINK1 and BNIP3) and NADH: Ubiquinone Oxidoreductase Subunit B8 (NDUFB8) and proved NDUFA6 gene expression. The tricarboxylic acid cycle is the main way for the body to obtain energy on the mitochondrial matrix. The results of qPCR showed that 3-PPA significantly inhibited the gene expressions of citrate synthetase (CS) and ketoglutarate dehydrogenase (OGDH) in the tricarboxylic acid cycle (Fig. 3g), along with the increase of acetyl-CoA content (Fig. 3h) and the acetylation level of total protein (Fig. 3i, j) in myotubes. To verify whether acetylation of protein mediates 3-PPA’s function, acetyltransferase inhibitor C646 was added to detect the myotube diameter, as shown in Fig. 3k, l, C646 was able to successfully reverse the effect of 3-PPA on promoting the myotube diameter. It’s reported that the NAD^+^/NADH ratio control the rate of tricarboxylic acid cycle, we found 3-PPA had a significant inhibitory effect on the NAD^+^/NADH ratio (Fig. 3m), and the mRNA expression of nicotinamide phosphoribosyltransferase (NAMPT) which is crucial to NAD^+^ synthesis (Fig. 3n). SIRT is a class of NAD^+^-dependent protein deacetylase, and 3-PPA also significantly inhibited the mRNA expression of SIRT1 and SIRT3 genes (Fig. 3o), consistent with the results of 3-PPA in accumulating the content of acetyl-coA in the C2C12 myotubes. Taken together, 3-PPA promotes acetyl-CoA content and acetylation modification by reducing the rate of tricarboxylic acid cycle and SIRT1/3 mRNA expression, which may suppress protein degradation and induce myotube hypertrophy.

### Foxo3/NAD^+^/Acetylation pathway is required for 3-PPA to increase protein acetylation modification.

**Fig. 4:**
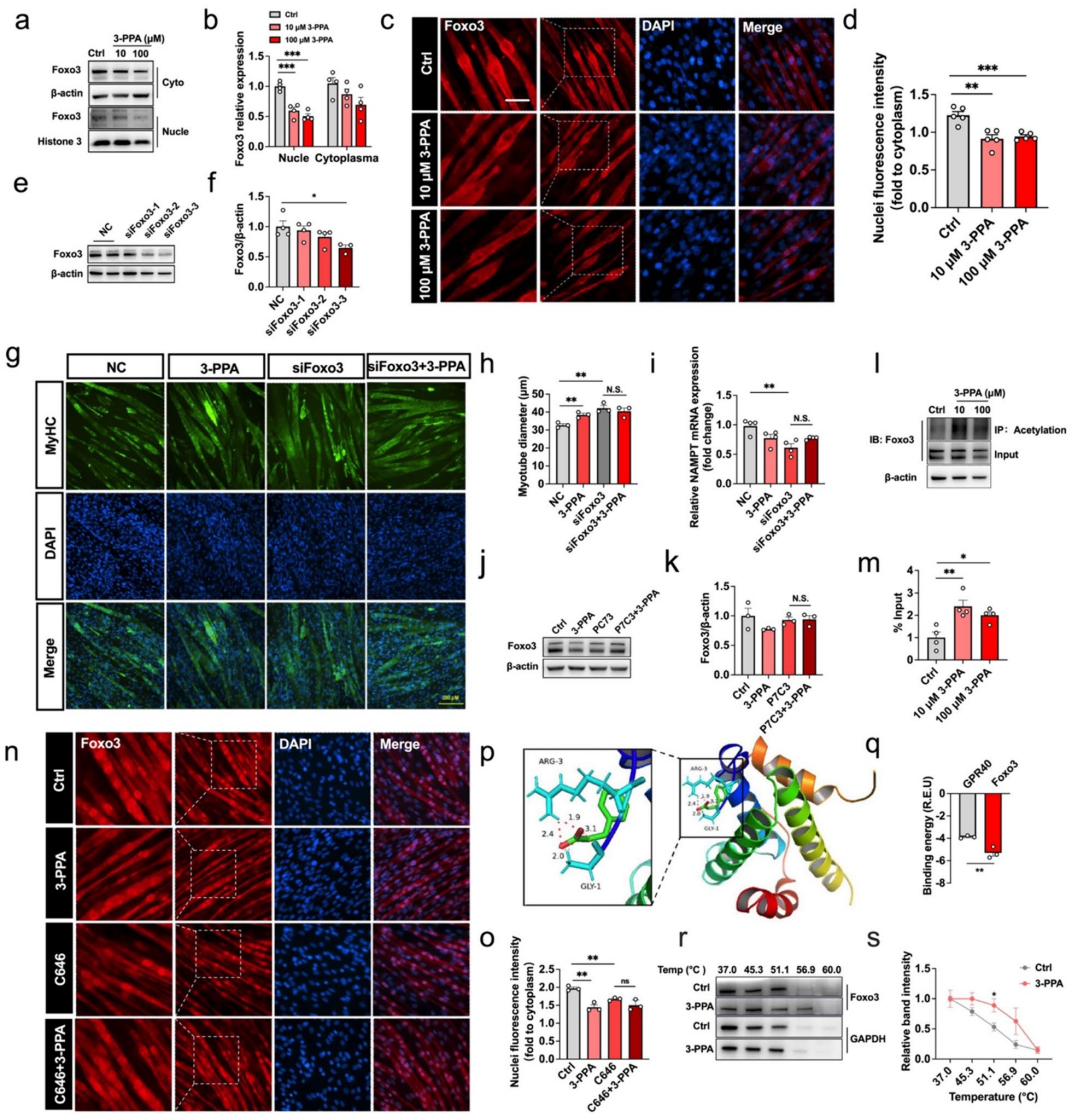
Foxo3/NAD^+^ pathway is required for 3-PPA to increase protein acetylation modification. **a–b** Immunoblots (**a**) and quantification (**b**) of Foxo3 protein expression in nuclei and cytoplasm of C2C12 myotubes with 3-PPA treatment for 2 days (n = 4). **c–d** Immunofluorescence images (**c**) and statistics (**d**) of relative nuclei Foxo3 protein in C2C12 myotubes in 3-PPA treatment for 2 days (n = 3). **e–f** Immunoblots (**e**) and quantification (**f**) of Foxo3 protein expression in C2C12 myotubes with different siRNA sequence treatment (n = 3 to 4). **g–h** Immunofluorescence images (**g**) and statistics (**h**) of myotube diameter in C2C12 myotubes with siFoxo3 and 3-PPA co-treatment (n = 3). **i** mRNA expression of NAMPT in C2C12 myotubes with si Foxo3 and 3-PPA co-treatment (n = 4). **j–k** Immunoblots (**j**) and quantification (**k**) of Foxo3 protein expression in C2C12 myotubes with P7C3 and 3-PPA co-treatment (n = 3). (**l-m**) Immunoprecipitation (**l**) and statistics (**m**) of Ace- Foxo3 in C2C12 myotubes with 3-PPA treatment for 2 days (n = 4). (**n–o**) Immunofluorescence images (**n**) and statistics (**o**) of Foxo3 of C2C12 myotubes in C646 and 3-PPA co-treatment (n = 3). (**p**) Binding region of 3-PPA and Foxo3 protein. (**q**) Calculated binding energy of Foxo3 and GPR40. **r–s** Immunoblots (**r**) and quantification (**s**) of relative Foxo3 protein expression in CETSA assay by 3-PPA treatment (n = 3)

**Fig. 5:**
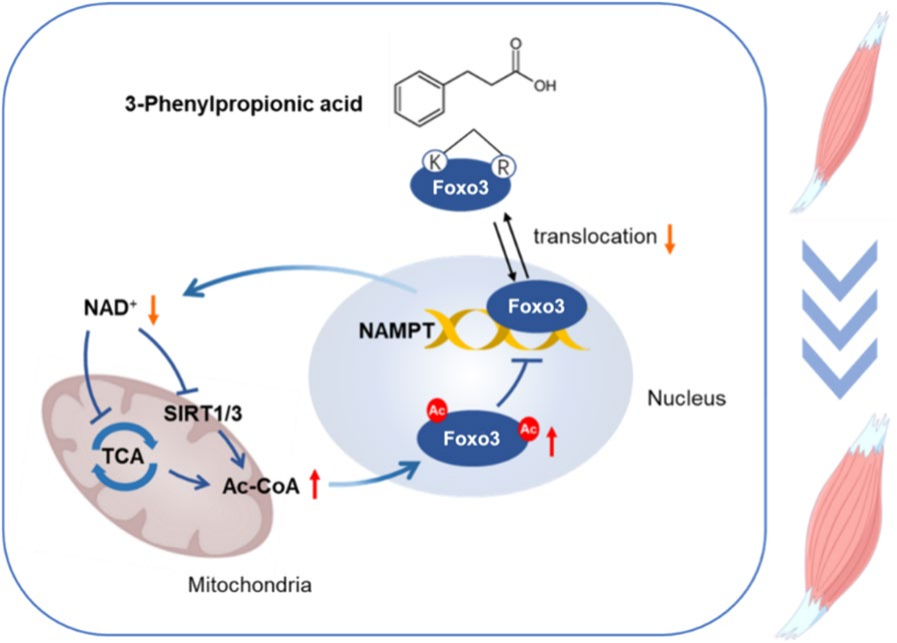
Schematic diagram of 3-PPA’s function on myotube hypertrophy

Further, we wonder if Foxo3 could correlated with actyl-CoA in playing the function of 3-PPA., Immunofluorescence analysis of Foxo3 revealed lower activity of the translocation between nuclei and cytoplasm (Fig. 4c, d), which is consistent with the decreased levels of nuclei and cytoplasm protein in immunoblotting result (Fig. 4a, b). In order to further verify the role of Foxo3 protein in the promotion of myotubes hypertrophy by 3-PPA, three siRNA sequences were used to interfere with Foxo3 protein expression, among which siRNA-3 has a significant inhibitory effect (Fig. 4e, f). After siRNA-3 treatment, the effect of 3-PPA on the myotube diameter disappeared (Fig. 4g, h). It has been shown that Foxo3 activity is directly regulated by NAD^+^ and SIRT1, and NAD^+^ synthesis related NAMPT gene is the target gene of Foxo3 protein at the same time. Consistently, we observed a decrease in NAMPT mRNA expression after knocking down Foxo3 expression (Fig. 4i). Acute activate of NAMPT activity with the specific agonist P7C3 significantly reverse the inhibitory effect 3-PPA in Foxo3 (Fig. 4j, k). What’s more, 3-PPA treatment triggered the acetylation of endogenous Foxo3 which causes inactive of Foxo3 protein (Fig. 4l, m), and acetyltransferase inhibitor C646 successfully altered this situation (Fig. 4n, o). Subsequently, we wonder if 3-PPA inhibits Foxo3 activity by directly binding. To figure this out, AutoDock 4.0 software was used to simulate the docking between 3-PPA and Foxo3 protein. The results showed that the calculated binding energy of 3-PPA and Foxo3 protein was significantly stronger than that of 3-PPA and GPR40 (positive control group) (Fig. 4p, q). And the CETSA result consistently proved the direct binding by increasing the thermal stability of Foxo3 protien (Fig. 4r, s). It is therefore tempting to speculate that 3-PPA promotes acetylation of Foxo3 by the coordinated sequential actions of NAD^+^ and SIRT1, and lower Foxo3 activity further inhibited NAD^+^ synthesis, which generated a positive feedback loop regulation. Hence, these data support the hypothesis that 3-PPA may inhibit the activity of Foxo3 by directly binding, thus inhibiting NAMPT mRNA expression, and promoting protein acetylation and myotube hypertrophy in skeletal muscle through NAD^+^/SIRT signaling pathway. Meanwhile, the NAD^+^/SIRT signaling pathway can further regulate Foxo3 protein expression (Fig. 5).

## Discussion

The development of skeletal muscle is a complicated biological molecular process which involves cell proliferation, differentiation and myotube hypertrophy [28], which mainly regulated by myogenic regulatory factors [29], muscle-specific miRNAs [30], and so on. Recent reports have highlighted the role of gut microbiota and its metabolites shed light on skeletal muscle function, composition, and development [12–15]. IPA, a bioactive metabolite of *C.sporogenes*, promotes C2C12 proliferation by regulating the myogenic regulatory factor signaling [18].Butyrate which produced by bacterial fermentation in the gut could alleviate skeletal muscle atrophy and enhance skeletal muscle mitochondrial function in the aging mice [31]. 3-PPA which is produced by *Firmicutes*, *Bacteroidetes* and *Actinobacteria* from phenylalanine (one of aromatic amino acids) in colon [17, 32] has few reports in regulating skeletal muscle development, and function.

In this study, we first indicated that different concentrations of 3-PPA significantly increased C2C12 myotubes diameter and promoted myotube hypertrophy, but failed to affect the proliferation and differentiation of C2C12 and repaired the muscle injury. So, why does 3-PPA only influence the hypertrophy of myotubes? According to the simplistic view, the key function factors of myogenic transcription factors were different [33–35] and the morphology of myotubes is not the same during myotubes normal differentiation. After 3 days’ differentiation when already existed primary myotubes [36], the muscle activity focus on the dynamically regulation of the protein synthesis and protein breakdown, which can be regulated by various molecules, such as AKT-mTOR, GPRs, ubiquitin-proteasome and so on [37, 38]. When 3-PPA added in whole process, we found the protein expression of MyoD and MyoG and the fusion index didn’t change. But in latest 2 day’s treatment, myotubes diameter significantly increased, and ubiquitination of total proteins inhibited. The distinct influence of 3-PPA in MRFs and protein degradation may take an explanation for its unique function in muscle hypertrophy. Also, the lack of detail study in proteasome and E1/E3 enzymes that are key biomarker in protein degradation needs complete in the future.

Energy metabolism of skeletal muscle affects the deposition and consumption of nutrients, regulating the skeletal muscle growth and development. Studies have found that secondary bile acids produced by intestinal microbial metabolism can activate downstream cAMP by binding with bile acid receptor 5 on skeletal muscle and increase skeletal muscle energy consumption [39]. Short-chain fatty acids can also participate in the transformation of skeletal muscle fibers from glycolysis to oxidation through regulation of AMPK activity, regulate the balance of body energy metabolism and improve endurance exercise ability [40]. Mitochondria are “energy factory”, its biological function and respiratory activity is related with TCA cycle in skeletal muscle which occurs in the mitochondrial matrix is the center of energy metabolism to connecting the metabolism of three major nutrients. The NADH and FADH2 produced by TCA cycle associated with the oxidative respiration transport chain to promote the generation of ATP from ADP [41], providing energy for the body's virtual activities. Our data showed that 3-PPA significantly decreased ATP content and NAD^+/^NADH ratio in the C2C12 myotubes, inhibited the gene expression of citrate synthase and ketoglutarate dehydrogenase, and restricted the TCA cycle. ATP has been regarded as an intracellular energy currency molecule in animal body, and the decrease of its content may indicate a vigorous stage of myotubes energy metabolism and a requirement for more ATP to consume. At the same time, we found that 3-PPA significantly inhibited the expression of genes related to mitochondrial autophagy but had no effect on the expression of genes related to mitochondrial fusion, generation and biological function. In conclusion, it is possible that 3-PPA can accelerate the growth rate of myotubes and increase the requirements of nutrient and energy. To maintain the normal level of ATP, PAKR2 and BNIP3 gene expressions are inhibited to reduce mitochondrial autophagy.

In addition to its important role of hydrogen and electron transfer in oxidation reduction and oxidative phosphorylation, NAD^+^ serves as a substrate for many metabolic enzymes, including SIRT, PARPs and DNA ligase [42].NAMPT, the rate-limiting enzyme in the NAD salvage pathway [43], plays a crucial role in maintaining skeletal muscle development and mitochondria function. Skeletal muscle specific NAMPT knockout mice display muscle atrophy, mitochondria dysfunction and more centralized nuclei [44]. However, when NAMPT protein levels are decreased by approximately 14%, it had no significant effect on the expression of mitochondrial respiratory capacity in skeletal muscle [45]. This study found that 3-PPA significantly inhibited the expression of NAMPT gene and the content of NAD^+^, but had no significant effect on the mitochondrial function in the C2C12 myotubes. Therefore, we suspect that 3-PPA might regulate the activity of some metabolic enzymes by inhibiting the synthesis of NAD^+^ to promote myotube hypertrophy rather than affecting mitochondrial oxidative phosphorylation and electron transport chains.

SIRTs, which use NAD^+^ as a co-enzyme, regulate protein activity, chromatin stability and gene transcription by catalyzing histone and non-histone deacetylation, and participate in physiological and biochemical processes in the body. The results of this study showed that 3-PPA significantly inhibits the synthesis of NAD^+^, and decreases the expressions of SIRT1 and SIRT3 genes in the myotubes. Therefore, we speculate whether 3-PPA affects myotube acetylation modification through the NAD^+^-SIRT signaling pathway. Protein acetylation is a post-translational modification of proteins, involving transfer the acetyl-coA and the ε-amino side chain under the action of acetyltransferase. It plays a regulatory role in stabilizing protein configuration, regulating protein activity and protein interactions. Studies have shown that the role of acetylation and deacetylation in the regulation of skeletal muscle mass may be both condition- and protein-specific [46]. And the acetylation of a lysine residue may block the binding of ubiquitin, thereby reducing the proteasome-dependent degradation of protein [47, 48]. Consistently, we found that 3-PPA significantly increased the content of acetyl-coA and promoted the acetylation modification of total protein in C2C12, while also leading to a decrease in the ubiquitination of total protein. And C646, an inhibitor of the histone acetyltransferase-CBP/p300 [49], successfully interdicted the effects of 3-PPA in myotube hypertrophy. Acetylation can play a role in the transcriptional expression of some myogenic regulatory factors. Both the acetyltransferase PCAF and p300 promote the acetylation of MyoD, thereby enhancing its transcriptional activity and promoting myoblast differentiation [50]. Pax7 can also be modified by the acetyltransferase MYST1 to regulate acetylation, which plays a key role in the activation of skeletal muscle satellite cells [51]. So next, we want to explore the target of the increasing acetyl-coA.

Foxo is involved in the regulation of cell cycle, metabolism, and protein turnover, and also affects protein ubiquitination [52] and NAD^+^-SIRT pathway. Some reports demonstrated that NAMPT gene can be targeted by Foxo for transcriptional regulation. Overexpression of Foxo1 can increase the transcription level of NAMPT by 62%, while knocking out Foxo1 reduced the transcription level of NAMPT by 30% [53]. NAMPT also is Foxo3 protein activity regulator, and up-regulation of NAMPT and SIRT1 activity is accompanied by an increase of Foxo3 protein level, which further regulates downstream gene expression [54]. SIRT1 is an important regulatory factor in the upstream of Foxo3. The activity of SIRT1 decreases after dephosphorylation, which affects the deacetylation modification of Foxo3 protein and thus reduces its activity [55], Additionally, the acetylation of the Foxo3 gene binding region can reduce its ability to activate target gene transcription [56]. Our data showed that 3-PPA significantly reduces Foxo3 protein expression and translocation to the nucleus by inducing its phosphorylation and acetylation. P7C3, an agonist of NAMPT, successfully reverse the effects of 3-PPA on Foxo3, and knockdown of Foxo3 significantly reduces NAMPT gene expression. By using AutoDock 4.0 software, we found 3-PPA can binding with Foxo3 protein directly.

## Conclusion

Based on our observation, we propose that 3-PPA may directly bind to Foxo3 protein, inhibit its activity, and consequently reduce the expression of NAMPT and SIRT gene. Through NAD^+^/SIRT signaling pathway, it promotes protein acetylation, competitively inhibits ubiquitination, and promotes myotube hypertrophy.

### Supplementary Information

Additional file 1: Fig S1 (a) OD value of CCK-8 to detect proliferation activity of C2C12 (n = 8). (b, c) EdU immunofluorescence images (b) and statistics (c) of proliferative activity of C2C12 cells (n=3). (d) Schematic representation of C2C12 treated with 3-PPA for 6 days. (e, f) Immunofluorescence images (e) and statistics of fusion index (f) of C2C12 myotubes in 3-PPA treatment for 6 days (n = 4). Fig S2 (a–c) Immunoblots (a) and quantification (b, c) of P-STAT3, STAT3, P-JAK2 and JAK2 protein expression of C2C12 myotubes with 3-PPA treatment for 0.5, 1, 3 and 6 h (n=3). (d–f) Immunoblots (d) and quantification (e, f) of P-mTOR, mTOR, P-AKT and AKT protein expression of C2C12 myotubes with 3-PPA treatment for 0.5, 1, 3 and 6 hours (n = 3). (g, h) Immunoblots (g) and quantification (h) of P-ERK and ERK protein expression of C2C12 myotubes with 3-PPA treatment for 0.5, 1, 3 and 6 h (n = 3). (DOCX 5570 KB)

## Data Availability

The data and materials that support the findings of this study are available in the methods of this article.

## Abbreviations

ADP: Adenosine diphosphate
AKT: Protein kinase B
ATG5: Autophagy related 5
ATG7: Autophagy related 7
ATP: Adenosine-5'-triphosphate
ATP5A1: ATP synthase alpha-subunit
BNIP3: BCL2 Interacting Protein 3
cAMP: Cyclic adenosine monoPhosphate
cDNA: Complementary DNA
COX6A1: Cytochrome c oxidase subunit 6a1
CT: Cycle threshold
CTX: Cardiotoxin
DEPC: Diethylpyrocarbonate
DMEM: Dulbecco’s modified eagle medium
DRP1: Dynamin-related protein 1
EDTA: Ethylenediaminetetraacetic acid
ERK: Extracellular signal-regulated kinase
FADH2: Flavin adenine dinucleotide
Foxo3: Forkhead Box O3
IGF-1: Insulin-like growth factor 1
JAK: Janus kinase
GAS: Gastrocnemius
GRP40: G-protein coupled receptor 40
GRP41: G-protein coupled receptor 41
LD: Lactic acid
mRNA: Messenger ribonucleic acid
mTOR: Mammalian target of rapamycin
MYHC: Myosin heavy chain
MyoD: Myoblast determination protein
MyoG: Myogenin
NAD^+^/ NADH: Nicotinamide adenine dinucleotide
NAMPT: Nicotinamide phosphoribosyltransferase
NDUFA6: NADH: ubiquinone oxidoreductase subunit A6
NDUFB8: NADH: ubiquinone oxidoreductase subunit B8
NMNAT2: Nicotinamide mononucleotide adenylyltransferase 2
PARK2: Parkinson protein 2
Pax3: Paired box 3
Pax7: Paired box 7
PBS: Phosphate buffered saline
PCR: Polymerase Chain Reaction
PGC1-α: Peroxisome proliferator-activated receptor gamma coactivator 1-alpha
PINK1: PTEN induced putative kinase 1
ROS: Reactive oxygen species
PVDF: Polyvinylidene Fluoride
SDH8: Succinate dehydrogenase subunit 8
SIRT1: Sirtuin 1
SQSTM1: Sequestosome 1
STAT: Signal transducer and activator of transcription
TBST: Tris Buffered Saline + Tween 20
TCA: Tricarboxylic acid cycle
TFAM: Mitochondrial transcription elongation factor
UQCRC1: Cytochrome b-c complex subunit 1
3-PPA: 3-Phenylpropionic acid
