# Supplementary material for: Microbiota-derived 3-phenylpropionic acid promotes myotube hypertrophy by Foxo3/NAD+ signaling pathway
Source: Cell Biosci. 2024 May 15;14:62. doi: 10.1186/s13578-024-01244-2 (PMC11097579; doi:10.1186/s13578-024-01244-2)
Supplement: Supplementary file 1 — Additional file 1: Fig S1 (a) OD value of CCK-8 to detect proliferation activity of C2C12 (n = 8). (b, c) EdU immunofluorescence images (b) and statistics (c) of proliferative activity of C2C12 cells (n=3). (d) Schematic representation of C2C12 treated with 3-PPA for 6 days. (e, f) Immunofluorescence images (e) and statistics of fusion index (f) of C2C12 myotubes in 3-PPA treatment for 6 days (n = 4). Fig S2 (a–c) Immunoblots (a) and quantification (b, c) of P-STAT3, STAT3, P-JAK2 and JAK2 protein expression of C2C12 myotubes with 3-PPA treatment for 0.5, 1, 3 and 6 h (n=3). (d–f) Immunoblots (d) and quantification (e, f) of P-mTOR, mTOR, P-AKT and AKT protein expression of C2C12 myotubes with 3-PPA treatment for 0.5, 1, 3 and 6 hours (n = 3). (g, h) Immunoblots (g) and quantification (h) of P-ERK and ERK protein expression of C2C12 myotubes with 3-PPA treatment for 0.5, 1, 3 and 6 h (n = 3). (DOCX 5570 KB) [file 13578_2024_1244_MOESM1_ESM.docx]

**Supplement** **Figures**

**
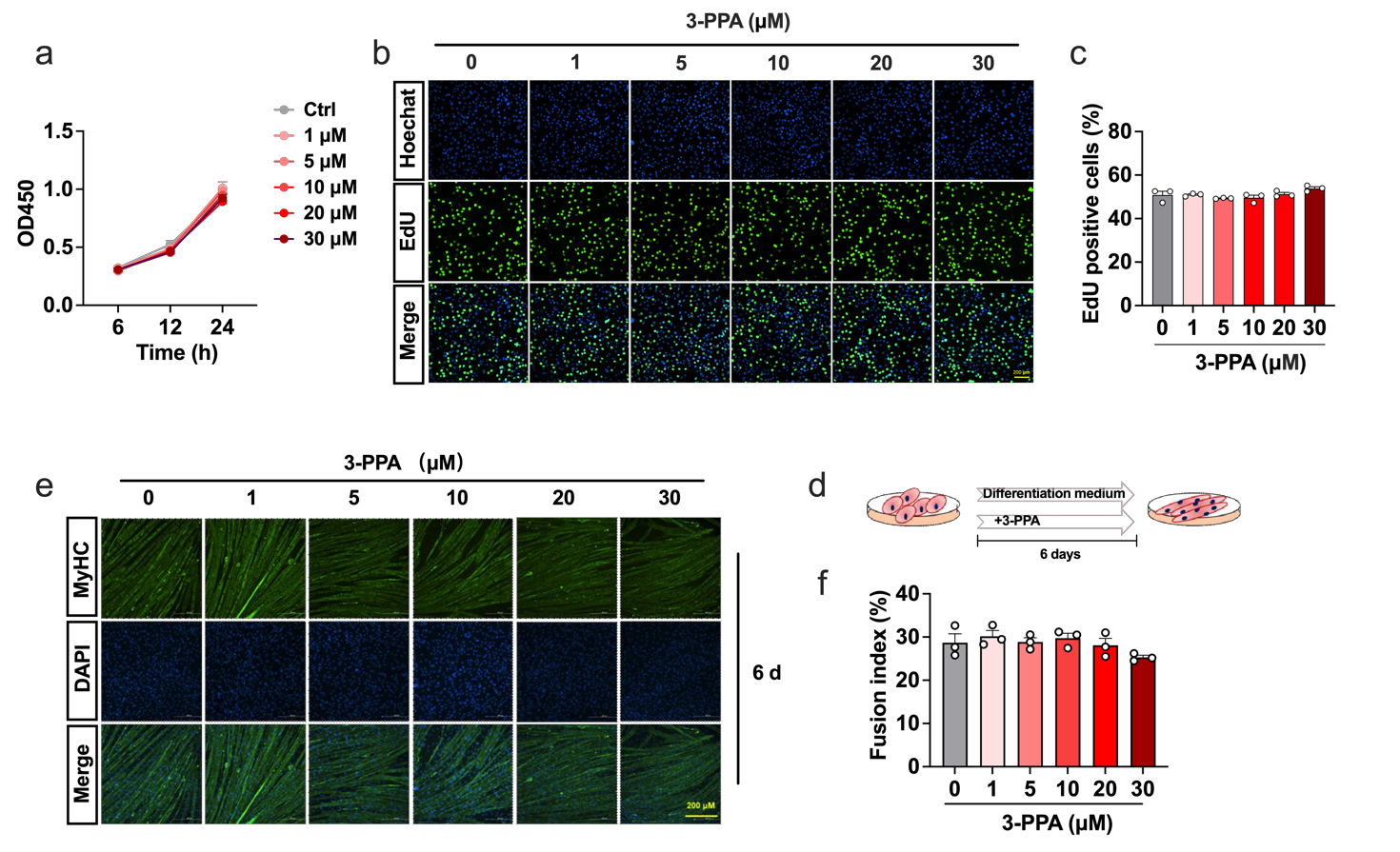
**

**Fig S1 (a)** OD value of CCK-8 to detect proliferation activity of C2C12 (n = 8). **(b-c)** EdU immunofluorescence images (b) and statistics (c) of proliferative activity of C2C12 cells (n=3). (**d**) Schematic representation of C2C12 treated with 3-PPA for six days. (**e-f**) Immunofluorescence images **(e)** and statistics of fusion index **(f)** of C2C12 myotubes in 3-PPA treatment for six days (n = 4).

**
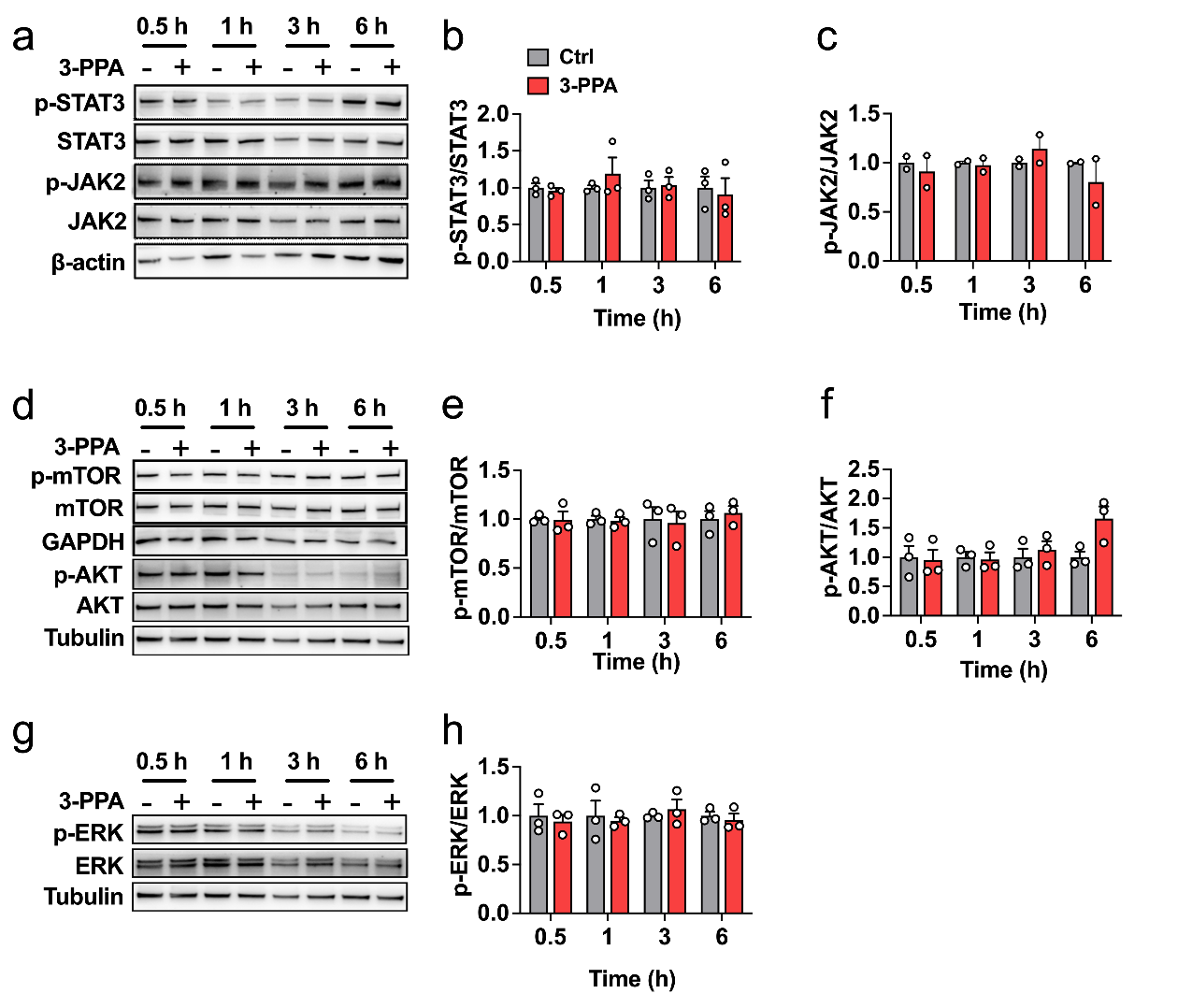
**

**Fig S2** (**a-c**) Immunoblots (a) and quantification (b-c) of P-STAT3, STAT3, P-JAK2 and JAK2 protein expression of C2C12 myotubes with 3-PPA treatment for 0.5, 1, 3 and 6 hours (n=3). (**d-f**) Immunoblots (d) and quantification (e-f) of P-mTOR, mTOR, P-AKT and AKT protein expression of C2C12 myotubes with 3-PPA treatment for 0.5, 1, 3 and 6 hours (n = 3). (**g-h**) Immunoblots (g) and quantification (h) of P-ERK and ERK protein expression of C2C12 myotubes with 3-PPA treatment for 0.5, 1, 3 and 6 hours (n = 3)
